# Preference for horizontal information in faces predicts typical variations in face recognition but is not impaired in developmental prosopagnosia

**DOI:** 10.3758/s13423-022-02163-4

**Published:** 2022-08-24

**Authors:** Zoë Little, Tirta Susilo

**Affiliations:** 1 School of Psychology, University of New South Wales, Sydney, Australia; 2 School of Psychology, Victoria University of Wellington, Wellington, New Zealand

**Keywords:** face perception and recognition, visual perception, perceptual categorization and identification

## Abstract

Face recognition is strongly influenced by the processing of orientation structure in the face image. Faces are much easier to recognize when they are filtered to include only horizontally oriented information compared with vertically oriented information. Here, we investigate whether preferences for horizontal information in faces are related to face recognition abilities in a typical sample (Experiment 1), and whether such preferences are lacking in people with developmental prosopagnosia (DP; Experiment 2). Experiment 1 shows that preferences for horizontal face information are linked to face recognition abilities in a typical sample, with weak evidence of face-selective contributions. Experiment 2 shows that preferences for horizontal face information are comparable in control and DP groups. Our study suggests that preferences for horizontal face information are related to variations in face recognition abilities in the typical range, and that these preferences are not aberrant in DP.

## Introduction

Face recognition is a complex function that involves multiple processing stages, beginning with lower-level processes that extract the face representation from the retinal image to higher-level processes that link the face representation to memory and knowledge. Face recognition research tends to focus on higher-level processes, but recent work shows that lower-level processes also make significant contributions. For example, face recognition is influenced by the location of the face in visual space (Afraz & Cavanagh, 2008; de Haas et al., 2016; Martelli et al., 2005), where people fixate on a face (Mehoudar et al., 2014; Peterson & Eckstein, 2013), and the tendency to fixate faces over objects (de Haas et al., 2019).

**Fig. 1:**
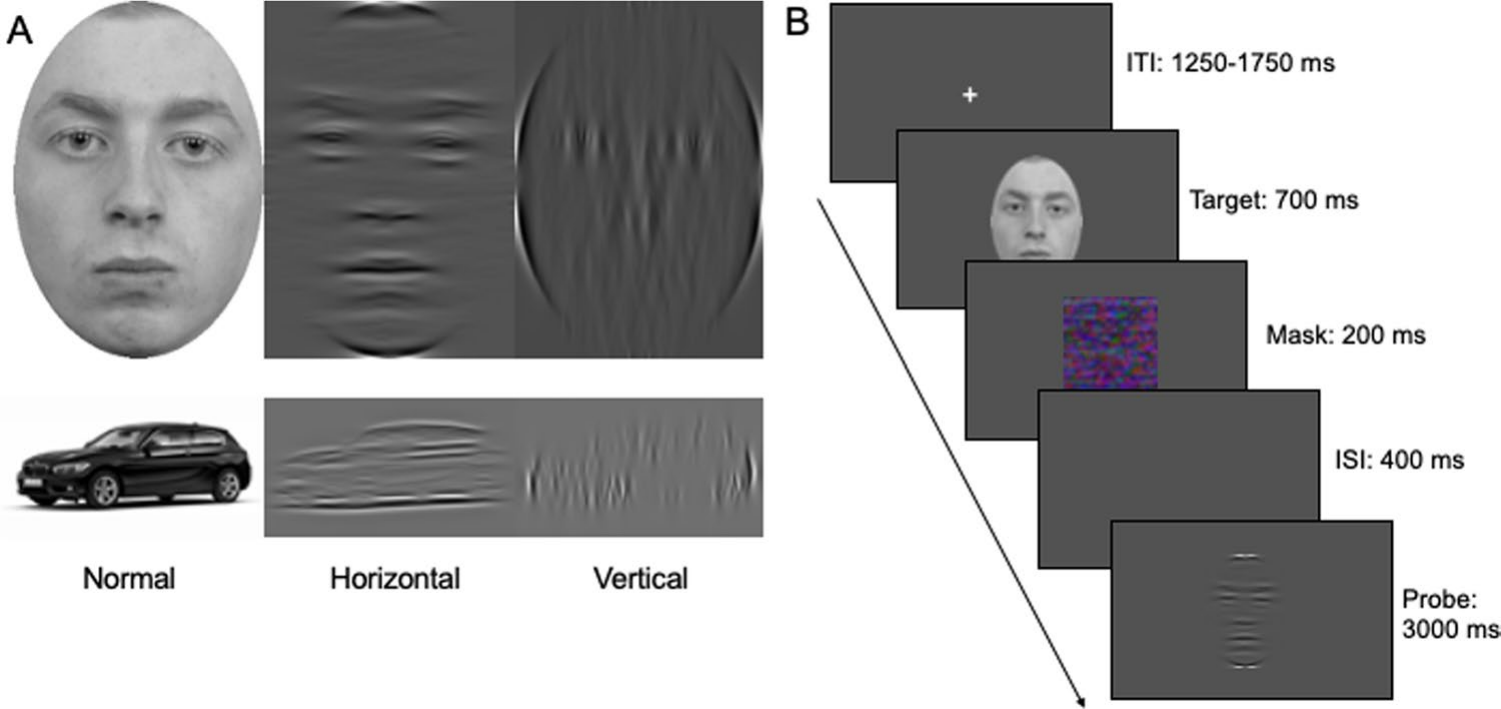
Face and car matching tasks. **A** Examples of one face and one car stimulus shown with each of the different filters. **B** An example trial in the face condition with a horizontal filter

A particularly important lower-level process in face recognition is the analysis of horizontal structure in the face image. Faces are much easier to identify when the face image is filtered to include only horizontal bands of information, compared with only vertical information (Fig. 1; Dakin & Watt, 2009; Goffaux & Dakin, 2010; Goffaux & Greenwood, 2016; Goffaux et al., 2015; Goffaux et al., 2011; Pachai et al., 2018; Pachai et al., 2013). Horizontal information is particularly useful for face recognition because it captures the arrangement of diagnostic features such as eyes and mouth, while vertical information carries less diagnostic information such as the edges of the head, the bridge of the nose, and the centre of the eyes (Dakin & Watt, 2009; Goffaux, 2019; Goffaux & Dakin, 2010; Goffaux & Rossion, 2007). Horizontal information also drives several hallmarks of face processing such as the inversion effect (Yin, 1969), the contrast negation effect (Galper, 1970), and the identity adaptation effect (Webster & Maclin, 1999). These effects emerge with horizontal information but not vertical information (Goffaux et al., 2011; Goffaux et al., 2015; Goffaux & Dakin, 2010; Pachai et al., 2013).

The importance of horizontal structure for faces is also demonstrated by individual differences work showing that preference for horizontal information in faces is linked to variations in face recognition abilities (Duncan et al., 2019; Pachai et al., 2013), with some evidence suggesting that the link may be face specific (Duncan et al., 2019). However, whether the link extends to extreme variations in face recognition abilities, such as in developmental prosopagnosia (DP), is unknown. Addressing this issue is important to better understand the role of horizontal structure in face processing, and to determine its contributions to face recognition skills in the typical and atypical range. We address this issue in two preregistered experiments, reported here (https://aspredicted.org/ny5u4.pdf). In both experiments participants had to decide whether two subsequently presented face images showed the same or different identities. The probe faces were filtered to include only horizontal or vertical information. Following previous studies (e.g., Goffaux & Dakin, 2010), we defined horizontal preference as performance with horizontal stimuli relative to performance with vertical stimuli.

In Experiment 1, we used an individual differences approach to ask whether horizontal preference for faces is linked to typical variations in face recognition skills as measured using the Cambridge Face Memory Test (CFMT; Duchaine & Nakayama, 2006). We did this by comparing the correlation between CFMT and horizontal preference for faces against the correlation between CFMT and horizontal preference for cars (as nonface control stimuli). We used cars to obtain a measure of general horizontal preference to complex objects since horizontal preference is not unique to faces (Dakin & Watt, 2009; Goffaux & Dakin, 2010). If what contributes to face recognition is general horizontal preference, then we would expect to see a similar correlation between CFMT and horizontal preference for either stimuli. But if horizontal preference for faces makes additional contributions to face recognition beyond any contributions from general horizontal preference, then we would expect to see a stronger correlation between CFMT and horizontal preference for faces than for cars.

In Experiment 2 we used a group-level analysis to examine whether horizontal preference for faces is impaired in developmental prosopagnosia (DP), the lifelong inability to recognize face identity (McConachie, 1976). We did this by comparing the size of the horizontal advantage (i.e., better performance for horizontal trials than for vertical trials) in a DP group against a sex/age-matched control group. If face deficits in DP are driven by an impairment of horizontal preference for faces, then the DP group should show reduced horizontal advantage for faces but not for cars relative to the control group.

## Experiment 1

### Method

#### Participants

Participants were 106 individuals (52 male, 51 female, three other; *M*_age_ = 33.47 years, *SD*_age_ = 8.43 years) from Testable Minds (www.minds.testable.org). This was the final sample size after 10 participants were excluded for performing below chance (50%) on either the face or car matching task (this is a less conservative threshold than what was preregistered, because overall accuracy was lower than anticipated). All participants gave informed consent and received US$4.50 for their participation. The experiment was completed online via Testable (www.testable.org; Rezlescu et al., 2020). Ethical approval was granted by the Victoria University of Wellington Human Ethics Committee.

#### Stimuli

Face stimuli were 15 frontal photographs of Caucasian male faces with neutral expressions from the Radboud face database. Each face was gray-scaled and cropped into an oval to remove external features such as hair and ears. Car stimuli were 15 black BMWs, a set of car stimuli we used previously (Rezlescu et al., 2016). Cars were photographed at an angle on a white background. Example stimuli can be seen in Fig. 1a. Cars were chosen for three reasons. First, cars share several theoretically important properties with faces—cars are real-world, three-dimensional objects that all share the same first-order structure (i.e., a body, wheels, doors, headlights, in a fixed relationship to each other; Diamond & Carey, 1986). Second, our pilot testing showed that cars have more diagnostic information in the horizontal orientation than in the vertical orientation, making cars suitable for testing horizontal preference. Finally, cars are commonly used as a comparison category in studies investigating face specificity (Barton et al., 2019; Dennett et al., 2012; Rossion & Caharel, 2011; Shakeshaft & Plomin, 2015). The images were matched in their low-level properties by equating the Fourier spectra of the whole images using the SHINE Toolbox implemented in MATLAB (Willenbockel et al., 2010). To create the filtered probe stimuli, the images were fast Fourier transformed, with the amplitude spectra multiplied with wrapped Gaussian filters (with a standard deviation of 14°) centred on 0° (vertical) or 90° (horizontal). The bandwidth of 14° was chosen to broadly match the orientation properties of V1 neurons (Blakemore & Campbell, 1969; Goffaux & Dakin, 2010; Ringach et al., 2002). The image processing script was created and provided by Goffaux and Dakin (2010). The first (target) images were presented at 280 × 400 pixels for faces and 400 × 218 pixels for cars. Viewing distance could not be controlled, as the experiment ran online; however, at a viewing distance of 60 cm, the size of the stimuli in degrees of visual angle would be 7.07° × 10.08° for faces and 10.08° × 5.51° for cars. The second (probe) images were presented at 75% of the size to reduce the contribution of lower-level retinotopic factors. A mask was created by scrambling of the unedited face images in GIMP 2.0. The image was repeatedly scrambled at the highest possible level until it resembled random noise.

#### Procedure

The procedure followed that of Goffaux and Dakin (2010). A fixation cross was presented for a random duration between 1,250 and 1,750 ms, then the first image was presented for 700 ms. The first image was always unfiltered. This was followed immediately by a mask the same size as the image for 200 ms. After a 400-ms interstimulus interval, the second image was shown for 3,000 ms. The position of the second image varied randomly from the first by ±20 pixels in the *x* and *y* dimensions. Participants could make their same or different response during or after the presentation of the second image by pressing either “s” or “d” on their keyboard. An example trial is shown in Fig. 1b.

Trials were split into four blocks: horizontally filtered faces, vertically filtered faces, horizontally filtered cars, and vertically filtered cars. Participants completed these blocks in a random order. Within each block the order of trials was randomized. Participants were able to take a break between blocks, and one break halfway through each block. Participants completed 60 trials per block (30 same, 30 different) for a total of 240 trials. The task took around 20 minutes to complete.

After the matching task, participants completed the Cambridge Face Memory Test (CFMT; Duchaine & Nakayama, 2006). Participants learnt six target faces by viewing each at one-third left/right profile and frontal view for 3,000 ms each, then completed three trials in which they indicated which face out of three was the one they just viewed. Memory for target faces was further tested across 54 trials in which participants identify one of the six targets from a line-up of three faces. Target faces were presented from new angles or in different lighting, or had Gaussian noise applied to them, ensuring that memory for the face was being assessed and not memory for the image. The CFMT took participants around 10 minutes to complete.

### Results

**Fig. 2:**
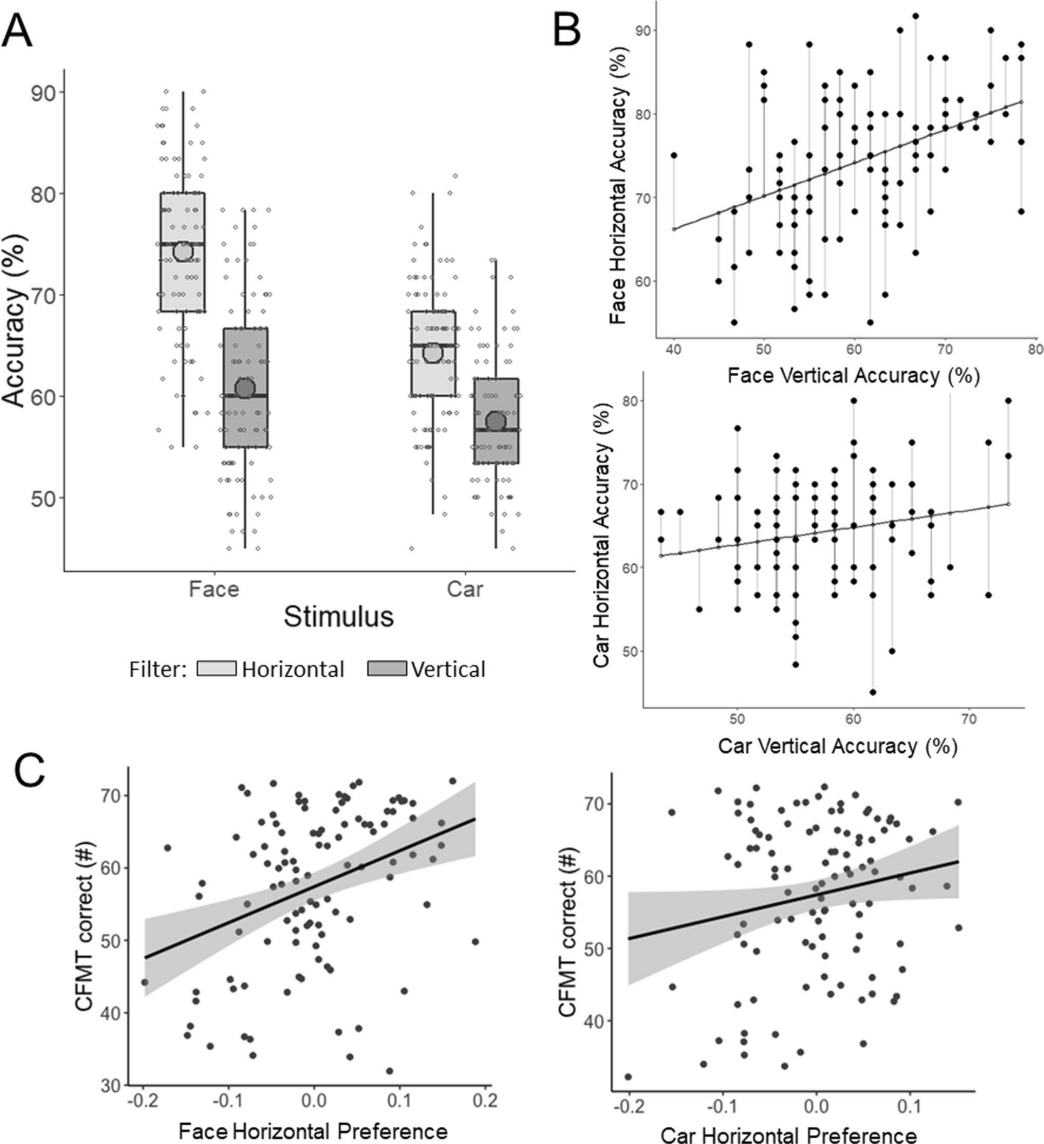
Results from Experiment 1. **A** Accuracy for horizontally- and vertically-filtered faces and cars. **B** How the regression-based measure of horizontal selectivity in face and car processing was calculated. **c** The correlation between CFMT and horizontal preference for faces (top) and cars (bottom) in a typical sample

Data for the first experiment were analyzed in jamovi 1.6 (The Jamovi Project, 2021). We first ran a 2 (stimulus: face, car) × 2 (filter: horizontal, vertical) repeated-measures ANOVA to check that the task yielded the expected group-effect at the group-level. A main effect of stimulus revealed higher accuracy for faces (*M* = 67.48%, *SD* = 7.22%) than for cars (*M* = 60.27%, *SD* = 5.11%), *F*(1, 105) = 86.18, *p* < .001, η_p_^2^ = .45. A main effect of filter revealed higher accuracy for horizontal stimuli (*M* = 69.29%, *SD* = 5.94%) than vertical stimuli (*M* = 58.91%, *SD* = 5.62%), *F*(1, 105) = 341.59, *p* < .001, η_p_^2^ = .77. These main effects were qualified by a significant interaction, *F*(1, 105) = 26.49, *p* < .001, η_p_^2^ = .20, whereby the advantage of horizontal over vertical stimuli was larger for faces (13.79%) than for cars (6.98%; Fig. 2a).

We next examined the link between horizontal preference and face recognition in the typical range. We quantified horizontal preference using a regression method (DeGutis et al., 2013), which is increasingly used in individual differences studies (DeGutis et al., 2012; DeGutis et al., 2014; Rezlescu et al., 2017). We fit a least squares regression line to the relationship between horizontal accuracy and vertical accuracy for faces and cars separately, with vertical accuracy as the predictor variable. We took the distance of each individual’s horizontal accuracy above or below the regression line (the residual) as the measure of horizontal preference because this measure captures variation in horizontal accuracy that is not accounted for by variation in vertical accuracy. This is represented in Fig. 2b. The regression method is advantageous because it ensures that variation in horizontal preference reflects variation in horizontal accuracy over and above variation in vertical accuracy, which has been statistically removed. With subtraction methods (i.e., when horizontal preference is computed by subtracting vertical accuracy from horizontal accuracy), variation in horizontal preference may result from variation in horizontal accuracy only, vertical accuracy only, or both at varying proportions, which cannot be teased apart.

Following our preplanned analyses, we correlated horizontal preference for faces and cars with the CFMT separately (Fig. 2c). Horizontal preference for faces was moderately correlated with CFMT, *r*(104) = .36, *p* < .001. Horizontal preference for cars was also correlated with CFMT, but the correlation was smaller and not significant (*r*(104) = .19, *p* = .052. However, these two correlations were not statistically different (*Z* = −1.43, *p* = .077, one-tailed), suggesting that what contributes to face recognition is general horizontal preference, although the numerically larger correlation with faces may indicate some face-specific contributions. For thoroughness, we also used the subtraction method, by subtracting vertical accuracy from horizontal accuracy as a measure of horizontal preference. There were no correlations between CFMT and horizontal preference for either faces, *r*(104) = .06, *p* = .512, or cars, *r*(104) = .02, *p* = .816.

We ran four non-planned analyses to provide a more complete and open examination of the data. First, we used multiple regression to test whether horizontal preference for faces predicts CFMT, controlling for horizontal preference for cars. The overall model was significant, *F*(2, 103) = 9.91, *p* < .001, and horizontal preference for faces predicted CFMT (β = 0.35, *p* < .001) after controlling for cars. Second, we used another regression approach, this time asking whether raw horizontal accuracy for faces predicts CFMT, controlling for raw horizontal accuracy for cars. We again obtained a significant correlation, *r*(104) = .46, *p* < .001. Third, we compared the basic correlations between CFMT and the raw measures. The correlation between CFMT and horizontal face accuracy, *r*(104) = .50, *p* < .001, was the largest and it was statistically different from the correlations between CFMT and horizontal car accuracy, *r*(104) = .23, *p* = .019; *Z* = 2.40, *p* = .008, or CFMT and vertical car accuracy, *r*(104) = .22, *p* = .025; *Z* = 2.46, *p* = .007. However, the correlation between CFMT and horizontal face accuracy is not statistically different from the correlation between CFMT and vertical face accuracy, *r*(104) = .42, *p* < .001; *Z* = 0.88, *p* = .190. Finally, we conducted a further multiple regression to see whether horizontal or vertical face accuracy was the better predictor of CFMT performance while controlling for variation associated with the other. The overall model was significant, *F*(2, 103) = 22.28, *p* < .001, and horizontal face accuracy (β = 0.39, *p* < .001) was a stronger predictor of CFMT score than vertical face accuracy (β = 0.26, *p* = .005). Overall, our planned and nonplanned analyses suggest that there is a preference for horizontal over vertical information in face processing. This preference predicts typical variations in face recognition abilities, with weak evidence of face-specific contributions.

## Experiment 2

### Method

Participants were 41 individuals with DP (*M*_age_ = 40.02 years, *SD*_age_ *=* 10.66 years, nine male, 31 female, one other) and 36 control participants (*M*_age_ = 37.72 years, *SD*_age_ *=* 8.95 years, 12 male, 23 female, one other). The control group was matched with the DP group on age, *t*(75) = 1.02, *p* = .312, and gender (χ^2^ = 1.29, *p* = .523). DP participants were recruited from the Prosopagnosia Research Centre (www.faceblind.org). Following our typical diagnostic procedure, DP participants scored two standard deviations below the control mean on the Prosopagnosia Index 20-item scale (Shah et al., 2015), the CFMT, and a famous faces test (Duchaine & Nakayama, 2005). Participants were excluded if they reported previous brain injuries or neurological disorders, or if their impaired scores on the Leuven Perceptual Organisation Test (Torfs et al., 2014) suggested broader deficits in basic visual processing. Control participants were recruited through Testable Minds (www.minds.testable.org).

The stimuli and procedure were the same as in Study 1, except DP participants did not complete the CFMT. Controls who scored in the clinical range of the CFMT (raw score <43) were excluded (*n* = 7) as they might have face recognition deficits but are unaware of them. The experiment was completed online via Testable (www.testable.org; Rezlescu et al., 2020). DP participants received a voucher for the equivalent of US$2.70 from their local Amazon store. Controls received US$4.50. All participants provided informed consent and ethical approval was granted by the Victoria University of Wellington Human Ethics Committee.

### Results

**Fig. 3:**
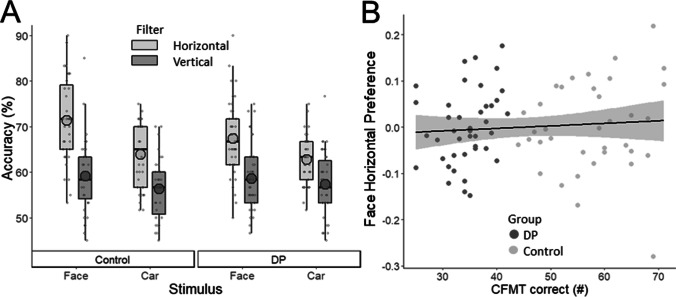
Results from Experiment 2. **A** Accuracy for the matching task in the DP and control groups. **B** Plot of the relationship between horizontal preference and CFMT scores in the DP (dark grey) and control (light grey) groups

Data for the second experiment were analyzed in jamovi (Version 1.6; The Jamovi Project, 2021) or JASP (Version 0.16.3; JASP Team, 2022; for Bayesian analyses). Results are shown in Fig. 3a. We compared performance between the groups with a 2 (group: control, DP) × 2 (stimulus: face, car) × 2 (filter: horizontal, vertical) mixed analysis of variance (ANOVA). Main effects of stimulus, *F*(1, 75) = 19.39, *p* < .001, η_p_^2^ = .21, and filter, *F*(1, 75) = 128.89, *p* < .001, η_p_^2^ = .63, revealed that accuracy was higher for faces (*M* = 63.76, *SD* = 7.04) over cars (*M* = 59.69, *SD* = 5.08), and for horizontal stimuli (*M* = 66.08, *SD* = 6.00) over vertical stimuli (*M* = 57.47, *SD* = 5.55). Similar to Experiment 1, a significant interaction between stimulus and filter, *F*(1, 75) = 4.06, *p* = .048, η_p_^2^ = .05, showed that the advantage for horizontal over vertical stimuli was larger for faces (10.19%) than for cars (7.03%). However, there were no significant main effects or interactions involving group (all *p*s > .050), and Bayes factors showed moderate evidence for these null effects (Group BF_incl_ = 0.17; Group × Stimulus BF_incl_ = 0.15; Group × Filter BF_incl_ = 0.25; Group × Stimulus × Filter BF_incl_ = 0.05). Bayes factors were calculated including all models. This result supports our interpretation that the horizontal advantage for faces was not reduced in the DP group.

## Discussion

We investigated the relationship between preference to horizontal information in faces and variations in face recognition skills in the typical range (Experiment 1) and in developmental prosopagnosia (DP) (Experiment 2). Experiment 1 shows that horizontal preference for faces and face recognition abilities in the typical range are correlated, but whether this correlation is selective to faces is unclear. The pre-registered analysis showed that the correlation is statistically comparable to a similar but numerically weaker correlation with cars, suggesting that the link between horizontal preference and face recognition abilities may not be face selective. In contrast, exploratory analyses suggest that preference for horizontal information in faces could explain this relationship over and above general horizontal preference. Experiment 2 shows that DP participants had similar preference for horizontal information in faces compared with controls. This result suggests that face recognition deficits in DP do not result from impairments in the processing of horizontal image structure. Overall, our study shows that preference for horizontal information in faces is related to typical variations in face recognition abilities, but it is not impaired in DP.

The moderate correlation between horizontal preference for faces and face recognition skills in a typical sample replicates previous findings (Duncan et al., 2019; Pachai et al., 2013), although our data indicate that the correlation may be less selective to faces than previously thought. Our finding of a weak correlation between horizontal preference for cars and CFMT suggests that some of this relationship may be explained by general preference for horizontal structure in any complex images (Dakin & Watt, 2009). Overall, our finding supports the notion that horizontal information is more important than vertical information for faces (Dakin & Watt, 2009; Goffaux et al., 2011; Goffaux et al., 2015; Goffaux & Dakin, 2010; Goffaux & Greenwood, 2016; Pachai et al., 2018) but also nonface objects (Dakin & Watt, 2009; Goffaux & Dakin, 2010).

Our study accords with growing literature showing that seemingly high-level face recognition skills are closely linked to a range of lower-level processes such as retinotopic processing (Afraz & Cavanagh, 2008; de Haas et al., 2016; de Haas et al., 2019; Martelli et al., 2005; Mehoudar et al., 2014; Peterson & Eckstein, 2013) and orientation structure analysis (Dakin & Watt, 2009). This literature suggests that the face recognition system may exploit the structure and function of early visual areas, such as oriented receptive fields and orientation tuning in V1 to help with the analysis of high-level face information (Goffaux & Dakin, 2010). Representing a face identity through the alignment of horizontal information would be advantageous as it would be resistant to changes in the viewpoint in which a face is seen from (Dakin & Watt, 2006; Goffaux & Dakin, 2010).

We also find that horizontal preference for faces is comparable in DP and control groups. This finding suggests that DP impairments do not result from a lack of horizontal preference in face processing. This finding also feeds into the ongoing discussion of whether people with and without DP differ in degree or kind, and whether DP is best viewed from a “quantitative” or “qualitative” standpoint (Barton & Corrow, 2016; Rossion, 2018). The quantitative view predicts that a linear relationship between face recognition skills and a variable of interest (i.e., horizontal preference) that is present in a typical sample would extend to those with DP, who occupy the low end of the face recognition spectrum (Barton & Corrow, 2016). As illustrated in Fig. 3b, DP participants showed greater horizontal preferences than what would be predicted by the linear trend in typical participants, making our finding more consistent with the qualitative view rather than the quantitative view. Moreover, this study illustrates the value of investigating whether DP deficits are linked to lower-level processes beyond the traditional focus on perceptual and memory processes. Such research may also yield insights into the relationship between lower-level and higher-level processes in human vision more generally, in typical and atypical brains.

The lack of impaired performance by the DP group in Experiment 2 may raise concerns about our task, but this is unlikely to be an issue for several reasons. First, our task is very similar in design to previous tasks (e.g., Goffaux et al., 2011; Goffaux et al., 2015; Goffaux & Dakin, 2010). Second, the task produced the expected horizontal advantage, with large effect sizes (*d* = 1.40 in Experiment 1, *d* = 1.00 in Experiment 2) similar to prior reports (e.g., *d* = 0.7 in Goffaux & Dakin, 2010). Third, the horizontal advantage across both experiments is consistently larger for faces than cars, indicating that our task is sensitive to face-specific processes. Fourth, task performance in Experiment 1 is correlated with CFMT, a well-validated measure of face recognition abilities. Finally, DP accuracy is far from floor and was statistically different to chance, *t*(40) = 13.21, *p* < .001. These reasons suggest that our task would have been sensitive enough to detect DP deficits, should they exist.

## References

[CR1] Afraz S-R, Cavanagh P (2008). Retinotopy of the face aftereffect. Vision Research.

[CR2] Barton JJS, Albonico A, Susilo T, Duchaine B, Corrow SL (2019). Object recognition in acquired and developmental prosopagnosia. Cognitive Neuropsychology.

[CR3] Barton JJS, Corrow SL (2016). The problem of being bad at faces. Neuropsychologia.

[CR4] Dakin SC, Watt RJ (2009). Biological “bar codes” in human faces. Journal of Vision.

[CR5] de Haas B, Iakovidis AL, Schwarzkopf DS, Gegenfurtner KR (2019). Individual differences in visual salience vary along semantic dimensions. Proceedings of the National Academy of Sciences.

[CR6] de Haas B, Schwarzkopf DS, Alvarez I, Lawson RP, Henriksson L, Kriegeskorte N, Rees G (2016). Perception and processing of faces in the human brain is tuned to typical feature locations. Journal of Neuroscience.

[CR7] DeGutis J, Cohan S, Mercado RJ, Wilmer J, Nakayama K (2012). Holistic processing of the mouth but not the eyes in developmental prosopagnosia. Cognitive Neuropsychology.

[CR8] DeGutis J, Cohan S, Nakayama K (2014). Holistic face training enhances face processing in developmental prosopagnosia. Brain.

[CR9] DeGutis J, Wilmer J, Mercado RJ, Cohan S (2013). Using regression to measure holistic face processing reveals a strong link with face recognition ability. Cognition.

[CR10] Dennett HW, McKone E, Tavashmi R, Hall A, Pidcock M, Edwards M, Duchaine B (2012). The Cambridge Car Memory Test: A task matched in format to the Cambridge Face Memory Test, with norms, reliability, sex differences, dissociations from face memory, and expertise effects. Behavior Research Methods.

[CR11] Diamond R, Carey S (1986). Why faces are and are not special: An effect of expertise. Journal of Experimental Psychology: General.

[CR12] Duchaine B, Nakayama K (2005). Dissociations of Face and Object Recognition in Developmental Prosopagnosia. Journal of Cognitive Neuroscience.

[CR13] Duchaine BC, Nakayama K (2006). Developmental prosopagnosia: A window to content-specific face processing. Current Opinion in Neurobiology.

[CR14] Duncan J, Royer J, Dugas G, Blais C, Fiset D (2019). Revisiting the link between horizontal tuning and face processing ability with independent measures. Journal of Experimental Psychology: Human Perception and Performance.

[CR15] Galper RE (1970). Recognition of faces in photographic negative. Psychonomic Science.

[CR16] Goffaux V (2019). Fixed or flexible? Orientation preference in identity and gaze processing in humans. PLOS ONE.

[CR17] Goffaux V, Dakin S (2010). Horizontal Information Drives the Behavioral Signatures of Face Processing. Frontiers in Psychology.

[CR18] Goffaux V, Greenwood JA (2016). The orientation selectivity of face identification. Scientific Reports.

[CR19] Goffaux V, Poncin A, Schiltz C (2015). Selectivity of face perception to horizontal information over lifespan (from 6 to 74 years old). PLOS ONE.

[CR20] Goffaux V, Rossion B (2007). Face inversion disproportionately impairs the perception of vertical but not horizontal relations between features. Journal of Experimental Psychology: Human Perception and Performance.

[CR21] Goffaux V, van Zon J, Schiltz C (2011). The horizontal tuning of face perception relies on the processing of intermediate and high spatial frequencies. Journal of Vision.

[CR22] The Jamovi Project. (2021). jamovi (Version 1.6) [Computer Software].

[CR23] JASP Team. (2022). JASP (Version 0.16.3) [Computer software].

[CR24] Martelli M, Majaj NJ, Pelli DG (2005). Are faces processed like words? A diagnostic test for recognition by parts. Journal of Vision.

[CR25] McConachie HR (1976). Developmental prosopagnosia. A single case report. Cortex.

[CR26] Mehoudar E, Arizpe J, Baker CI, Yovel G (2014). Faces in the eye of the beholder: Unique and stable eye scanning patterns of individual observers. Journal of Vision.

[CR27] Pachai MV, Bennett PJ, Sekuler AB (2018). The bandwidth of diagnostic horizontal structure for face identification. Perception.

[CR28] Pachai M, Sekuler A, Bennett P (2013). Sensitivity to information conveyed by horizontal contours is correlated with face identification accuracy. Frontiers in Psychology.

[CR29] Peterson MF, Eckstein MP (2013). Individual differences in eye movements during face identification reflect observer-specific optimal points of fixation. Psychological Science.

[CR30] Rezlescu, C., Chapman, A., Susilo, T., & Caramazza, A. (2016). Large inversion effects are not specific to faces and do not vary with object expertise. *PsyArXiv*. 10.31234/osf.io/xzbe5

[CR31] Rezlescu C, Danaila I, Miron A, Amariei C, Parkin BL (2020). More time for science: Using Testable to create and share behavioral experiments faster, recruit better participants, and engage students in hands-on research. *Progress in brain research* (Vol. 253, pp. 243–262).

[CR32] Rezlescu C, Susilo T, Wilmer JB, Caramazza A (2017). The inversion, part-whole, and composite effects reflect distinct perceptual mechanisms with varied relationships to face recognition. Journal of Experimental Psychology: Human Perception and Performance.

[CR33] Rhodes G, Brake S, Atkinson AP (1993). What’s lost in inverted faces?. Cognition.

[CR34] Rossion B (2018). Prosopagnosia? What could it tell us about the neural organization of face and object recognition?. Cognitive Neuropsychology.

[CR35] Rossion B, Caharel S (2011). ERP evidence for the speed of face categorization in the human brain: Disentangling the contribution of low-level visual cues from face perception. Vision Research.

[CR36] Shakeshaft NG, Plomin R (2015). Genetic specificity of face recognition. Proceedings of the National Academy of Sciences.

[CR37] Susilo T, Godfrey HK (2019). Prosopagnosia without object agnosia? A systematic study of a large sample of developmental cases. Journal of Vision.

[CR38] Towler J, Gosling A, Duchaine B, Eimer M (2012). The face-sensitive N170 component in developmental prosopagnosia. Neuropsychologia.

[CR39] Webster MA, Maclin OH (1999). Figural aftereffects in the perception of faces. Psychonomic Bulletin & Review.

[CR40] Willenbockel V, Sadr J, Fiset D, Horne GO, Gosselin F, Tanaka JW (2010). Controlling low-level image properties: The SHINE toolbox. Behavior Research Methods.

